# Political Assassination

**Published:** 1900-08-11

**Authors:** 


### The Hospital

A Journal of

The Medical Sciences and Hospital Administration.

Vol. XXVIII.—No. 724.] Edited by Sir Henry Burdett, K.C.B., and Solomon C. Smith, M.D., M.R.C.P. [August 11, 1900.

### POLITICAL ASSASSINATION.

The recent murder of the lamented King of Italy, together with the attempts made upon the lives of the Prince of Wales and of the Shah, and with the statements attributed to members of the so-called "Anarchist" societies in various parts of the world, appear to show that the crime of political assassination has entered upon a new phase, of such a kind as to call for the serious attention of all civilised nations, not only as to overt acts which may be prevented, but also as to the mental conditions in which such acts have their origin. The assassins of Royalty in bygone times, such men as Clement or Ravaillac, or Gérard, were generally pure fanatics, whose hatred was limited to the selected victim, and had been called into existence by some reality, by an act which he had done, or had threatened to do, or had omitted doing. They had no accomplices, and no hostility to monarchy in the abstract; but hoped for the attainment of something which they thought good, or for the prevention of something which they thought evil, by the removal of a single personage who stood in the way of their desires. In our own day, the aim of the political assassin seems to be to destroy the roots of Government by striking impartially at any of its heads; an undertaking in which the inadequacy of means to ends would appear, to persons of ordinary capacity, so plainly manifest as to point to grave disturbance of mental equilibrium on the part of any who could seriously engage in it. In the lowest decadence of the Roman Empire, no one was ever hindered from assuming the purple by the consideration that its wearers were inevitably short-lived; and, under the conditions of modern civilisation, the legitimate successor ascends at once to the vacant throne, and the machinery of government pursues its course undisturbed. The marvel is that persons should exist who think that assassinations can in any important degree modify the course of human events, and who are so far possessed by this delusion as to be ready to sacrifice their own lives in order to take but one step towards the attainment of the objects which they profess to desire. None, even of those who most abhor the crimes, can deny that the criminals are at least earnest in their convictions; and the question which presses for solution is how these convictions are brought about. By what inherited vice of intellect, by what perversion of education, or under the influence of what vicious propaganda does it befall that men who are apparently rational can expect to ameliorate the condition of mankind by the murder of their rulers. Causes of some kind must be in active operation; and it seems foolish to neglect them, and to deal only with their effects.

Why do people believe anything? and, especially, why do they believe numbers of things as to which they have no evidence? "There are multitudes," wrote Faraday, "who think themselves competent to decide, after the most cursory observation, upon the cause of this or that event (and they may be really very acute and correct in things familiar to them); a not unusual phrase with them is, that 'it stands to reason' that the effect they expect should result from the cause they assign to it, and yet it is *very difficult*, in numerous cases that appear plain, to show this reason, or to deduce the only true and rational relation of cause and effect." As far as can be ascertained, the mentally uneducated majority of mankind will believe anything which is asserted to them with sufficient positiveness and iteration, without the slightest regard either to its probability or to the evidence on which it rests. Fashionable ladies, for example, will believe in the cruelty and wickedness of experimentation on living animals; and hundreds or thousands of well-meaning working men believe, more or less, in the silly and venal rant of the "leaders" who flourish in idleness upon their labour. It can hardly be matter for surprise that Italians, descendants of a race which has lived for centuries under the intellectually paralysing influence of the Papacy, come to believe in the dogmatically expressed opinions of such a man as this Malatesta, who is said to be the present high priest of the sect of the assassins, and who, in all probability, is simply a dangerous lunatic, with sufficient cunning to provide for the preservation of his own skin.

Statesmen cannot rightly remain indifferent to, or unobservant of, the currents of prevailing popular delusions which, sooner or later, will be translated into action; and it is far better to anticipate crime than to punish it after it has been committed. The preaching of anarchist doctrines, and the advocacy of assassination as a means of promoting their spread, should be regarded as sufficient ground for the permanent incarceration of the preacher in an asylum for the incurably insane. Such a fate would not be likely to call forth imitators, and would strike at the root of the delusion. The actual assassins should, of course, be hanged whenever or wherever they could be captured; but this is a comparatively unimportant detail. The root of the whole matter is in insanity; and, pending the time when this becomes amenable to science, it should unquestionably be made amenable to discipline.

